# Challenges to Implementing an Environmental Flow Regime in the Luvuvhu River Catchment, South Africa

**DOI:** 10.3390/ijerph16193694

**Published:** 2019-09-30

**Authors:** Pfananani Ramulifho, Esther Ndou, Reuben Thifhulufhelwi, Tatenda Dalu

**Affiliations:** 1 SARCHI Chair in Biodiversity Value and Change in the Vhembe Biosphere Reserve Research Group, University of Venda, Thohoyandou 0950, South Africa; 2 Centre for Water Resources Research, University of KwaZulu-Natal, Scottsville 3209, South Africa; ndou.estelle@gmail.com; 3 Environmental Learning and Research Centre, Rhodes University, Grahamstown 6140, South Africa; thanyani.reuben@gmail.com; 4 Aquatic Systems Research Group, Department of Ecology and Resource Management, University of Venda, Thohoyandou 0950, South Africa; dalutatenda@yahoo.co.uk

**Keywords:** catchment management, environmental flows, Luvuvhu Rivers, stream flow, water allocation, water use

## Abstract

Rivers are now facing increasing pressure and demand to provide water directly for drinking, farming and supporting industries as a result of rapidly growing global human population. Globally, the most common practice for catchment managers is to limit water abstraction and changes to stream flow by setting environmental flow standards that guard and maintain the natural ecosystem characteristics. Since the development of the environmental flow concept and methods in South Africa, very few studies have assessed the institutional constraints towards environmental flow implementation. This study determined stream flow trends over time by fitting simple linear regression model to mean daily stream flow data at three selected stations in the Luvuvhu River Catchment (LRC). We also conducted a literature search to review, firstly the response of aquatic organisms (fish and macroinvertebrate) to changes in habitat conditions and secondly on local challenges affecting the sustainable implementation of environmental flow regime and related water resources management strategies. All the three stream flow stations show decreasing stream flow volume of 1 and 2 orders of magnitude faster in some stations with the possibility that flow will cease in the near future. Qualitative analyses from both local and international literature search found that the main challenges facing the implementation of sustainable flow strategies and management are absence of catchment management agency, lack of understanding of environmental flow benefits, limited financial budget, lack of capacity and conflict of interest. Rivers with changing stream flows tend to lose sensitive species. The development of scientifically credible catchment-wide environmental flow and abstraction thresholds for rivers within the LRC would make a major contribution in minimizing the declining stream flow volumes. Monitoring and reporting should be prioritized to give regular accounts of the state of our rivers.

## 1. Introduction

River scientists are challenged by the question of how much water a river needs to maintain its natural health and to sustain the integrity of aquatic and riparian ecosystems. With growing pressure and demand on aquatic ecosystems to provide water directly for drinking, agriculture and supporting industries and domestic use, the alteration of natural stream flow conditions is probably the biggest threat to the integrity of aquatic ecosystems [1,2,3,4,5]. Conflicts over the most essential services of aquatic ecosystems particularly rivers, between ecological and societal needs are increasing as global population and water demand rises [6,7]. Surface water resources over southern Africa, especially river flow, have been projected to decrease by up to 35% by 2050 in rivers of the Limpopo catchment which incorporates the Luvuvhu catchment as a result of ongoing climatic change [3,6,8]. Reduction in stream flow may lead to a change in perenniality of rivers from permanent to non-perennial, while permanent wetlands are becoming seasonal or temporary.

A consensus exists within the scientific community that rivers need to maintain and resemble the natural variation of flow conditions to sustain the ecological health and array of goods and services they provide to society [1,2,9,10]. In recent times, water regulations have progressed to account for stream flow regimes that effectively protect freshwater ecosystems [10,11]. This is because stream flow regime shapes many fundamental ecological characteristics of riverine ecosystems [12,13]. For example, rivers are classified as having excellent habitat structure based on their flow, and its resultant thermal and geomorphology signatures [14]. All elements of a flow regime (i.e., magnitude, frequency, duration, timing and rate of change) are important in structuring aquatic communities [1,2,15,16]. Regardless of all these advances in generating trade-off strategies, which advocates for alternative and ecological water use scenarios, water policy and management have been implemented comparatively slowly. Thus, environmental flow regimes in rivers are currently being implemented in only a tiny fraction both at regional and catchment scales worldwide, with a vast majority of management applications only focusing on low flow [9,17].

Stream flow strategies that meet human needs while preserving and maintaining the ecological functioning of river systems have been passed into law in the South African National Water Act, through the declaration of the “ecological reserve” [18]. The goal behind the ecological reserve is to negotiate for satisfactory trade-offs in water allocation among all users of the resource and the resource base itself (the river). Since the development of the environmental flow concept and methods in South Africa [6,19], very few studies have assessed the institutional constraints to environmental flow implementation. Therefore, a sound evaluation of constraints to stream flow management should be based on specific information concerning management challenges and the implication between flow changes and ecological response [20].

The aims of this study were to (1) determine the stream flow regime trend and anticipated impacts on aquatic organisms in the Luvuvhu River catchment, Limpopo province, South Africa; and (2) review local challenges affecting the sustainable management of river flows, especially in relation to the institutional capacity of the river management framework to provide environmental flows. We reviewed river management issues reported in the literature and contextualized them against an institutional capacity of Luvuvhu River management framework. This study also discusses the general ecological impacts of declining flow regimes and provides key management actions that can assist in overcoming stream flow management challenges.

## 2. Methods

### 2.1. Study Area

**Figure 1:**
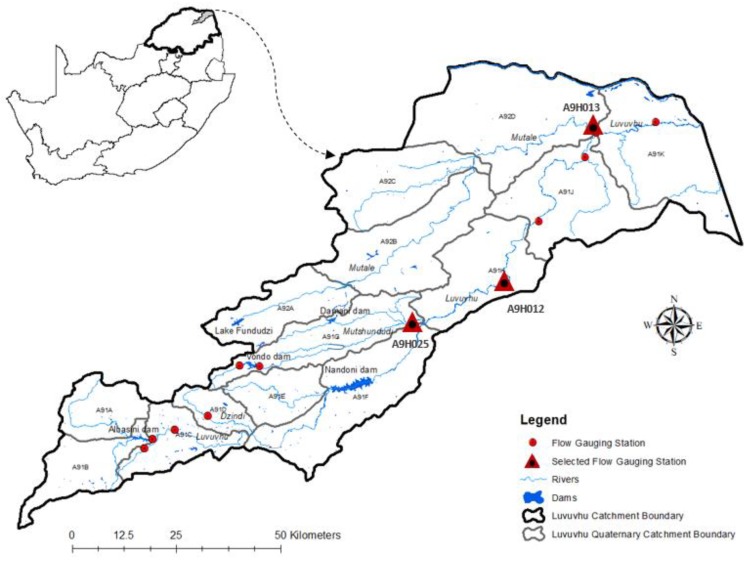
Map of the Luvuvhu River catchment highlighting the major dams, gauging stations and quaternary catchments.

We studied the catchment management of the Luvuvhu River catchment (LRC), Limpopo province, South Africa (Figure 1). This catchment is currently subjected to major forms of stream flow alteration and its rivers play a significant role for both the human and ecological elements [21]. Long-term annual rainfall and air temperature has not changed over the last 100 years in both the lower (Kruger National Park) and the upper (Levubu) sections of the Luvuvhu catchment (Figures S1 and S2). Other studies in the LRC recognize the need to limit both water abstractions and changes to the river flow regimes by setting environmental standards that guard and maintain river ecosystem characteristics and functions [22,23]. The Luvuvhu River catchment is located in the Luvuvhu–Letaba Water Management Area, with a catchment size of approximately 5941 km^2^ (Figure 1), with 14 quaternary catchments, with the main river channels being the Luvuvhu, Mutale, Mutshindudi and Dzindi Rivers [23,24]. The Luvuvhu River catchment is important to forestry, macadamia, avocado and banana plantations in the headwaters, agricultural development in the valleys and lower slopes (western side) and rural and urban settlements (eastern side) [25], while the rivers and the riparian vegetation of the Luvuvhu catchment are home to many bird and animal species [26].

Elevation in the catchment varies from 1587 m in the west, where the Soutpansberg Mountains are located, to about 200 m in the eastern section of the catchment where the Luvuvhu River joins the Limpopo River [27]. The catchment climate is considered semi-arid, with most of the rainfall occurring during summer, and is largely driven by the topography of the area [25]. The mean annual temperature varies from 20 °C in the western section to 22 °C in the eastern section [28]. The mountainous areas of the LRC receive the highest mean annual precipitation of 1800 mm as compared to 400 mm in the lowland areas. However, the mean annual precipitation for the entire catchment varies between 610 to 800 mm [24,27], with a mean annual runoff of 519 million m^3^ [27]. The variability of streamflow has increased within the Luvuvhu River Catchment over an 86-year period, indicating that factors such as anthropogenic activities and reservoir development could be impacting on the river streamflow [22,29]. Changes in stream flow regimes will affect the poor whose households’ livelihood activities are water-dependent.

### 2.2. Flow Data and Trend Analyses

Stations with long-term (>20 years) flow data that include the most recent flow periods were selected across the catchment (Table 1), with stations monitoring canals, pipeline and sluice gates being excluded from the analyses. Stations were inspected for completeness of stream flow data records and statistical anomalies. Gaps longer than one month were excluded from the analyses. We kept these stations in the analyses to account for best spatial variability. Only stream flow data was studied because it provides strong surrogacy for human impacts on stream flow changes and can be used to make deduction on climate change [30].

We fitted linear regression models to mean daily stream flow time series data using simple linear regression (SLR) to model temporal trends at three selected stations. A positive value of the slope indicates an increasing trend and a negative value a decreasing trend. Linear regression models and patterns in the temporal trends were run and visualized using the ‘lm’ and ‘ggplot’ function in R, respectively [31].

### 2.3. Literature Search and Analysis

A search was conducted on the Web of Science and Google Search for local and international literature on stream flow regimes and water management strategies for the years between 1939 and 2019. In total, 480 publications were identified and those which were closely related to the topic and peer-reviewed were included. We excluded studies that focused only on ecological management, ecological impact and water quality that did not relate to water and catchment management, respectively [32].

## 3. Results

### 3.1. Stream Flow Changes

**Figure 2:**
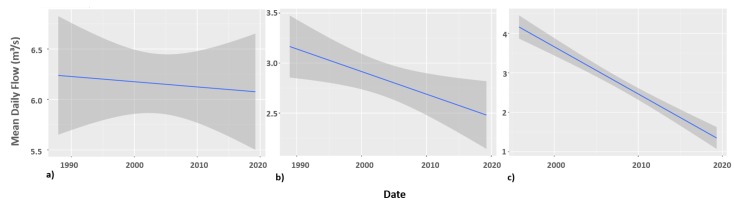
Fitted regressions of time series of streamflow at (**a**) Mhinga, (**b**) Kruger and (**c**) Matsika in the Luvuvhu catchment. Grey ribbons represent 95% confidence intervals for streamflow.

Figure 2 shows the linear regression results for Mhinga, Kruger and Matsika stream flow stations. All the three stream flow stations are highlighting a decreasing trend in stream flow volume (Figure 2; Table 2). These negative trends in long-term streamflow regime are significant for Kruger (*p* = 0.016) and highly significant for Matsika (*p* < 0.001). Stream flow volume is decreasing at 1 and 2 orders magnitude faster at Matsika than at Kruger and Mhinga stations (Figure 2). The figure shows that the mean daily stream flow trends in Matsika and Kruger are more likely to cross the zero flow intercept in near future (around 2030) than flow in Mhinga. This is because the greatest decreases in stream flow volume are rapidly occurring in small sub-catchments.

### 3.2. Possible Impacts on Aquatic Organisms Resulting from Changing Flow Regimes

Numerous studies have examined how aquatic organisms respond to changes in the quantity of water in river catchments around the world. Some dynamics on how fish and macroinvertebrate species are affected by changes in flow components is presented in Table 3. Modification of the natural flow regime has affected aquatic species in aquatic ecosystems worldwide, and the greater the departure from the natural regime the greater the loss of organisms and their ecosystem services [2,33,34]. Some studies have reported that disturbed patterns of stream flow events possess critical implications for aquatic organisms, particularly fish and macroinvertebrate species, that determine their relative success and regulate ecosystem process rates [12,35]. Studies have highlighted that the majority of fish and macroinvertebrate responses involved losing sensitive species as a result of altered flow magnitude when water flow decreases [12]. Reduction in stream flows in rivers and subsequent drying leads to rapid loss of fish and macroinvertebrates diversity as the habitat disappears (Table 3) [12,13]. Both increase and decrease in flow magnitude generally decreases macroinvertebrate and fish abundance and diversity in rivers. Rivers with physical disturbance from floods (and droughts) will have unstable substrates and tend to be characterized by low species diversity, and the biota found in such disturbed rivers often have life histories or behavioural characteristics of frequently disturbed environments [1,33,36,37].

### 3.3. Challenges Facing the Implementation of Environmental Flows

Of the 480 local and international papers reviewed on stream flow regimes and water management strategies, 432 (90%) reported natural flow regimes, methods of setting environmental flow standards, ecological consequences of altered flow regimes, management strategies and a wide range of political, economic, social and environmental circumstances surrounding water resources. Only 48 papers (10%) reported on terrestrial research and management that lack relevancy to stream flows and catchment management and were not included in this study. Five prominent challenges were found, which are: (i) absence of catchment management agencies/authorities (CMA), water user associations and water boards, (ii) lack of understanding of environmental flow benefits, (iii) limited financial budget, legal position and technical hydrological resources, (iv) lack of institutional and human capacity and (v) conflict of interest. These challenges facing sustainable stream flow management and the related river ecology have been evident within the LRC since the 1960s [27,46,47]. It is also apparent that declining stream flow is progressively degrading both the water quantity and associated fauna and flora within the catchment [20].

#### 3.3.1. Absence of Catchment Management Agencies (CMA), Water User Associations, and Water Boards

The absence of a catchment specific water resource governing structure in many situations is the baseline challenge in resource management. In the LRC situation, this causes the South Africa Department of Water and Sanitation (DWS) regional office to be the primary manager of the water resource [27]. Legislative issues, human and financial resources limit the scope for many water management agencies operate as a water resource management body, as some policies are silent on water resource conservation and protection. It is only the CMA (in cooperation with water user associations and water boards) which is directed through the National Water Act, to collaboratively protect, allocate, conserve, manage and control water resources within a specific catchment [48,49]. The main aim of setting up catchment management agencies is to decentralize responsibility for managing water resources so that water users and the public at large can play their part in managing and conserving the resource [48,49]. Currently, it is now close to two decades since the encouragement for the formation of the water users association (which include municipal, industrial, agricultural and domestic users) and CMA within the LRC [26]. No functioning structures are in place, making it hard to implement environmental flow management strategies and educate users on sustainable resource use.

#### 3.3.2. Lack of Understanding of Environmental Flow Benefits

The importance of environmental flows in sustaining ecosystem services, local economies and other river-dependent organisms is still largely unrecognized and under-appreciated within the LRC [26], while primary water uses for domestic and agricultural purposes still enjoy the highest priority. Very little environmental flow related benefits were recognized during the environmental and social impact assessment phases that were undertaken in 1997 for the then-proposed dams in the Luvuvhu and Lutanandwa Rivers [27]. Environmental flows are perceived by many as more restrictive and political, serving only for officials who benefit from water resource management, as opposed to seeing them as developmental and conservation tool [4,5,46,50]. The widespread perception is that the impact of large water–resource developments on riparian communities is little understood, with continued misperception that environmental flows are intended to benefit primarily non–human species [50,51]. Riparian communities prefers stream flow over reservoirs, exhausting all of the low flows in the river, particularly in the critically dry period of August to November, making it hard to implement sustainable stream flow actions in the LRC [29]. Because of this, several studies suggest that the existing and potential impacts of aquatic resource loss are high [4,5,50,52,53]. As long as communications about environmental flows remain centred on non-human benefits and conspicuously absent in the public media, these misperceptions of ecological reserves benefits will persist and it will be difficult to implement and conserve them [10].

#### 3.3.3. Limited Financial Budget, Legal Position and Technical Hydrological Resources

The challenges associated with the development and implementation of the LRC standards for environmental flow include financial, hydrological resources and legal constraints [49]. The way a CMA, water user association and/or water board is financed is of great significance for its success within the sustainable management of the catchment at stake [26,49], with potential to decelerate implementation of related water policies and procurement of critical and required hydrological information equipment [26,28,49]. The absence of operating legal binding rules for dams in the LRC causes sanctioning of high priority action with small financial allocation [26]. Inadequate technical tools for maintaining instream flow have been reported as huge constraints towards fully operating ecological reserves within the LRC [23,25].

#### 3.3.4. Lack of Capacity

A lack of capacity in terms of human resources in the government, with numbers being particularly low relative to the size of the system, limits performance [25,49]. Shortage of dedicated environmental or hydrological staff and relevant departments results in staff having to double up on their responsibilities and placement in positions for which they are not adequately trained [54]. Coupled with limited management capacity, CMAs are unable to undertake and achieve their delegated mandates [49].

## 3.3.5. Conflict of Interest

The conflicting logjam for a priority use of water between major water supply schemes in upper catchments (e.g., Albisini government water scheme, Vondo regional water supply scheme and Luvuvhu irrigation scheme), sewage treatment works and the Malamulele regional water supply scheme in the lower catchment need to be harmonized within the LRC [27]. It is also common that environmental flow management in many jurisdictions, regulation of surface water, groundwater and dams are not coordinated; therefore, action areas conflict and/or overlap [10]. Due to lack of drinking water delivery in rural areas, infrastructure development and health, the government is further crippled by pressure to allocate adequate funds amongst these critical operational areas to eradicate backlogs in service delivery [10]. Water managers are also hesitant to release bulk water for environmental purposes when other human uses could be jeopardized [10]. Thus, implementing environmental flows in an unstable environment is a complex task, with conflict exacerbated by the need to recover water currently being used for other important purposes such as agriculture, industry and domestic supply, possibly bringing unintended economic and social impacts [55].

## 4. Discussion

### 4.1. Declining Stream Flow and Aquatic Organisms

This study found that natural flow volumes are currently declining significantly across the LRC, with the trend suggesting that flow is likely to cease in the foreseeable future. The decrease in mean flow volumes is apparently a human-driven process, since the decline is not consistent with the climatic conditions of the region (Figures S1 and S2). Rainfall and temperature in the Luvuvhu catchment have remained unchanged over the last 100 years [56]. Other studies in the LRC [20,22] have reported the rate of water abstraction for commercial forestry and agricultural purposes as an immediate concern for the sustainable future of stream flow regimes. Though our analysis was unable to place flow alteration in a more specific environmental context, several studies have shown that aquatic organisms are more likely to respond negatively to changes in hydrologic drivers [11,12,40]. Catchment management strategies should acknowledge natural flow as the cornerstone in catchment management strategies [57], hence aiming to preserve the natural flow in rivers for the protection of natural freshwater ecosystems’ biodiversity and the human livelihood and well-being that depend on these ecosystems [7,14,58]. In general, changes in stream flow regimes in the Luvuvhu catchment would not be favourable for the aquatic organisms and people who depend on this catchment for water. From a South African perspective, there is a huge potential for sustainable conservation of the LRC, given the simplicity of adopting widely applied tools such as Downstream Response to Imposed Flow Transformations (DRIFT) [59]. DRIFT is a scenario-based approach that predicts and attempts to avoid severe impacts to flow, aquatic organisms and socio-economic factors of proposed water-resource developments on rivers [50].

### 4.2. Sustainable Catchment Management Prospects for the LRC

The main problem in the LRC is the absence of proper management institutions and strategies to address issues such as water allocation and conservation through an integrated management approach. Appropriate and capable river management institutions are needed and should be established within the LRC. These sentiments have been suggested by other studies done in this catchment [20,28]. River managers should set environmental flow regime standards and thresholds to achieve specific conservation objectives and support a diverse range of organisms, which otherwise is unattainable without stream flow management strategies [9,60]. Environmental flows should be set to resemble the natural flow regime of the river, thus enabling indigenous flora and fauna communities to prosper and provide their share of ecosystem services and benefits to humans [50,57]. The choice for a best method to implement environmental flow regimes in catchment management is limited to merits and types of issues to be addressed [6,61,62]. This is always influenced by a hydrological statutory authority (catchment management strategies or approach) to address water resources challenges and objectives [35], as recommended following the process detailed in a white paper [34] and by Department of Water Affairs and Forestry (DWAF) [26]. The national DWS recognizes that CMAs hold an important responsibility in hydrological landscape management, as they capture and synthesize essential water resource information as well as its strategic implementation for a sustainable future. The performance of these agencies that have a responsibility for the monitoring and managing river ecosystems in terms of their allocation of resources, implementation of actions and achievement of goals should continuously be evaluated and updated in a timely manner [26,63].

Implementation of sustainable catchment management strategies requires proper governance systems that ensure that CMAs gain adequate understanding of catchment water availability and land use impact within their water management areas [36]. To enhance stakeholders (local communities) engagement, environmental flow priority-setting and related benefits from their application should be communicated from a political and socio-economic water resource perspective [32]. A transparent, inclusive and well–coordinated stakeholder dialogue helps to popularize the notion that mankind does benefit from environmental and ecological flow regimes through a number of important silent services such as tourism, fishing, water retention and storage, and water supply and purification [34,36]. Therefore, as such, CMAs can then constrain human impacts on natural flow variability by better monitoring water withdrawal permits and dam licensing [32,35,36].

Several approaches can be adopted to aid in conflict resolution and mediation skills, developing capacity, and raising funds towards implementation of environmental flow standards at catchment levels. Catchment conflicts over stream flow trade-off can be settled using a structured rational approach that values future goals and needs over perceptions, while harmonizing and mediation through the Water Tribunal and Courts of Law, as these institutions play an important role where serious water disputes arise. Institutional capacity to sustainably manage a LRC can be developed by raising awareness, identifying resources and capacity requirements to achieve compliance, collaboration with other catchment CMAs, increasing access to related environmental flow and ecosystem related information, and outsourcing capacity and resources that are available nationally for building capacity and assisting CMAs in achieving their overall targets [33,34,35,37,64]. An assessment of how the actual river health meets the stated vision, goals and objectives and the specific management actions that have been set should be carried out regularly [26]. The CMAs can raise funds through water–use (e.g., increasing tariffs for higher use and time-limited water abstraction licenses) and waste discharge charges and external financial investments [35].

## 5. Conclusions

The development of scientifically credible catchment-wide flow management strategies and abstraction thresholds for rivers within the LRC would make a major contribution in minimizing the declining stream flow volumes due to water abstraction. Stakeholders will benefit from the biodiversity and essential ecological goods and services provided by the LRC ecosystem, while maximizing economic and social welfare in an equitable manner. The LRC will provide for human well-being in multiple ways, especially among rural poor communities living close to the river system [24,33]. The implementation of these scientifically credible catchment guidelines for the protection and restoration of ecological integrity of rivers will squash all misperceptions that environmental flow thresholds are intended to benefit primarily fauna and flora [10,35]. For successful water resource management, it will always be necessary to have an understanding of how people and climate influence the distribution of daily flows. Monitoring and reporting should be prioritized to give regular accounts of the state of our rivers. Such a management exercise will mark the start of better policing of the LRC water resources and aid in moving towards the implementation of environmental flow strategies.

## Figures and Tables

**Table 1:** Description of stream flow stations used in the study.

Flow Station	Location	River	Lat (°S)	Long (°E)	Upstream Dam	Period	Period (Years)	Data Completeness (%)	Catchment Area (km²)
A9H012	Mhinga	Luvuvhu	−22.86	30.88	Nandoni	1987–2019	32	95.4	1758
A9H013	Kruger	Mutale	−22.43	31.07	None	1988–2019	31	75.7	1776
A9H025	Matsika	Mutshundudi	−22.85	30.68	Thathe	1996–2017	23	78.0	387

**Table 2:** Statistical results for flow trends analyses from the three selected stations flow. Trend significance level is indicated by * *p* < 0.05; ** *p* < 0.01.

Station	Y (Intercept)	Slope	Standard Errors	*p* Value
Mhinga	6.33	−1.413 × 10^−5^	4.476 × 10^−5^	0.750
Kruger	3.59	−6.185×10^−5^	2.556 × 10^−5^	0.016 *
Matsika	7.25	−3.281×10^−4^	2.945 × 10^−5^	<0.001 **

**Table 3:** Summary of the main changes in ecological responses of fish and macroinvertebrate organisms in relation to the changes in the elements of streamflow regimes.

Changes in Flow Regime	Fishes	Macroinvertebrate
Magnitude	Alteration of flow results in loss of sensitive species [12]. Fish migratory changes both upstream and downstream due to too little water that impedes fish movement [38]. The magnitude of flood peaks can determine the degree of scouring mortality of fish egg [11].	Alteration of flow results in loss of sensitive species [12]. Greater magnitude of extremes causes life cycle disruption [12]. Desiccation of macroinvertebrates [39].
Frequency	High frequency of flow, non-native species of fish may fail to establish [11]. Decreased reproduction and abundance of the native fishes [40]. Influences the reproduction and mortality events of various species [41]. Decreased richness of endemic and sensitive species [40]. Fish migratory changes such as increased upstream migration [42].	Increasing frequency of high flow disturbances, macroinvertebrate communities shift toward species adapted to high mortality rates, such as those having short life cycles and high mobility [11]. Increased variation results in life cycle disruption [2].
Duration	Reducing the duration of low flows would not be expected to have a large effect on native fish [11]. Increase in abundance of non-native species [36]. Increasing the duration of low flows could dewater habitat and damage native species [11].	Decreased duration of floodplain inundation causes loss of floodplain specialists in mollusc assemblage [12]. Increasing the duration of low flows would limit habitat available for invertebrate assemblages [39,40].
Timing	The natural timing can prevent the establishment of non-native fish [11]. Loss of seasonal flow peaks disrupts cues for fish: spawning [2].	Reduced survivorship of larval atyid shrimps following early summer spates [1]. Human-induced changes in timing may cause productive failure, stress and mortality [41].
Rate of Change	The loss of seasonal flooding can promote success of non-native fish species [11]. Fish stranding and drifting [43,44].	Accelerated flood recession results in failure of seedling establishment [2]. Macroinvertebrate drift [45].

## Supplementary Materials

The following are available online at https://www.mdpi.com/1660-4601/16/19/3694/s1, Figure S1: Unchanging long-term patterns of (a) annual and (b) monthly rainfall (mm) of Kruger climate records (MacFadyen et al., 2008), Figure S2: Unchanging rainfall in relation to minimum and maximum temperatures in the Levubu area.
