# Supplementary material for: Challenges to Implementing an Environmental Flow Regime in the Luvuvhu River Catchment, South Africa
Source: Int J Environ Res Public Health. 2019 Sep 30;16(19):3694. doi: 10.3390/ijerph16193694 (PMC6801372; doi:10.3390/ijerph16193694)
Supplement: Supplementary file 1 [file ijerph-16-03694-s001.pdf]

International Journal of
Environmental Research and Public Health

Supplementary Material

Challenges to Implementing an Environmental Flow Regime in the Luvuvhu River Catchment, South Africa

Pfananani Ramulifho [1,*], Esther Ndou [2], Reuben Thifhulufhelwi [3] and Tatenda Dalu [4]

1. SARCHI Chair in Biodiversity Value and Change in the Vhembe Biosphere Reserve Research Group, University of Venda, Thohoyandou 0950, South Africa
2. Centre for Water Resources Research, University of KwaZulu-Natal, Scottsville 3209, South Africa
3. Environmental Learning and Research Centre, Rhodes University, Grahamstown 6140, South Africa
4. Aquatic Systems Research Group, Department of Ecology and Resource Management, University of Venda, Thohoyandou 0950, South Africa

* Correspondence: pfananani.ramulifho@gmail.com

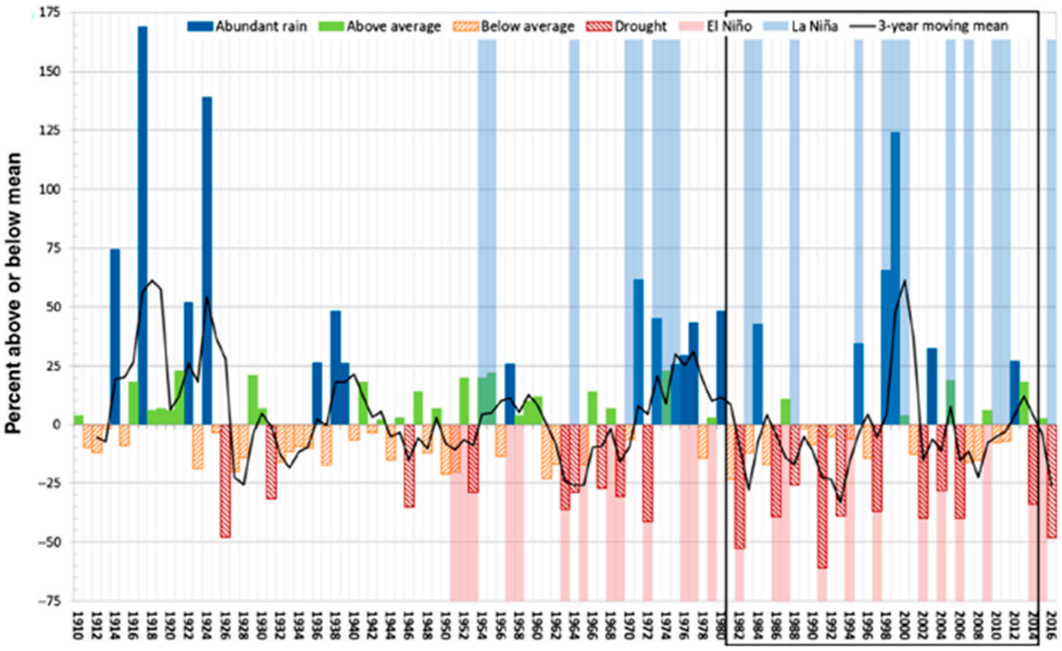

Fig. S1. Unchanging long-term patterns of annual rainfall (mm) of Kruger climate records [57].

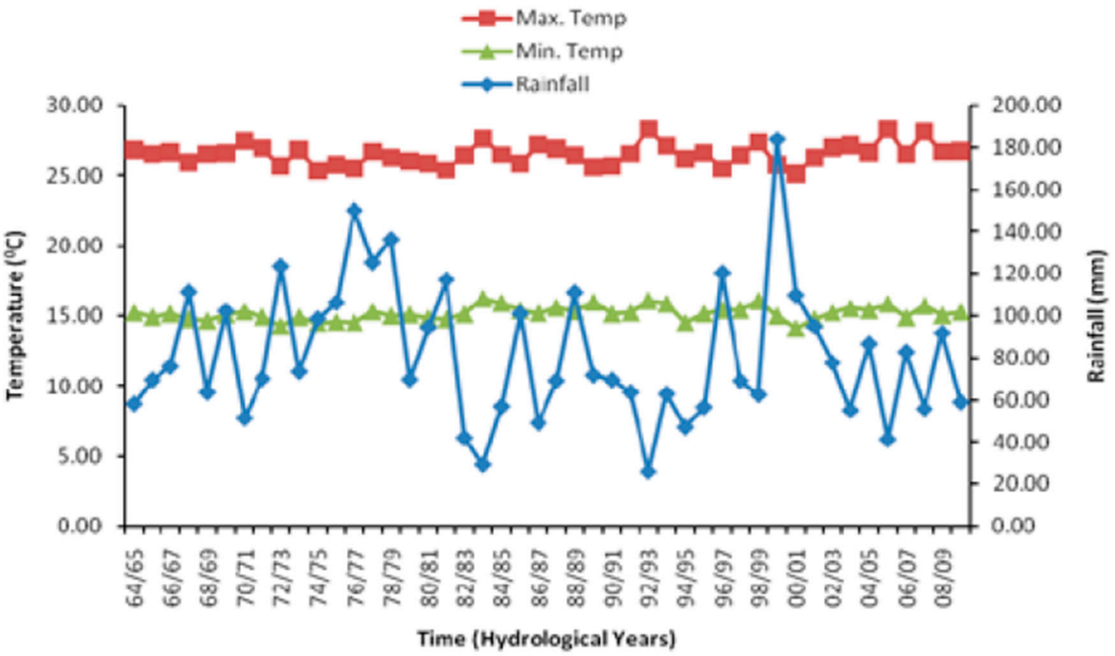

Fig. S2. Unchanging rainfall in relation to minimum and maximum temperatures in the Levubu area.

© 2019 by the authors. Submitted for possible open access publication under the terms and conditions of the Creative Commons Attribution (CC BY) license (http://creativecommons.org/licenses/by/4.0/).
